# Are seronegative patients with rheumatoid arthritis and clinically suspect arthralgia properly represented in randomized clinical trials?

**DOI:** 10.1007/s10067-024-07187-w

**Published:** 2024-10-26

**Authors:** Bernardo D’Onofrio, Carlo Selmi, Elisa Gremese

**Affiliations:** 1 Rheumatology and Clinical Immunology, IRCCS Humanitas Research Hospital, Rozzano, Milan Italy; 2 Department of Biomedical Sciences, Humanitas University, Pieve Emanuele, Milan, Italy

**Keywords:** Rheumatoid arthritis, Clinically suspect arthralgia, Autoantibodies, ACPA, Randomized controlled trials

## Abstract

Rheumatoid arthritis (RA) is a chronic immuno-inflammatory disease whose outcomes can vary greatly from one patient to another. One of the main prognostic factors is the presence of serum autoantibodies, such as rheumatoid factor (RF) and anti-citrullinated peptide antibodies (ACPA). Indeed, when seropositive, patients with RA are at higher risk of radiographic progression, disability, and increased mortality. Moreover, while the introduction of the 2010 American College of Rheumatology/European Alliance of Associations for Rheumatology (ACR/EULAR) classification criteria has allowed for an earlier diagnosis, studies on large early arthritis cohorts have also shown that these criteria are less capable of identifying seronegative patients, who are therefore at a higher risk of being diagnosed and treated late. In light of these, the major randomized controlled trials have mostly enrolled patients with autoantibody-positive disease. However, in recent years, it became evident that the two serotypes of RA differ significantly from many points of view. Alongside this, a greater understanding of the disease pathogenesis, particularly the presence of antibodies in patients’ serum even before the onset of arthritis, has generated significant interest in exploring whether the disease could be prevented by treating patients in the pre-arthritis phases. Once again, emerging trials predominantly enroll subjects positive for RA autoantibodies, potentially overlooking seronegative individuals with arthralgia-at-risk.

The management of rheumatoid arthritis (RA) has substantially improved during the last decades, thanks to the earlier diagnosis and prompt initiation of the disease-modifying therapy within the “window of opportunity” and the subsequent monitoring according to a treat-to-target regimen aimed at remission. Moreover, an increasing number of available molecules have been tested in patients with RA [1], resulting in a broad therapeutic armamentarium and better outcomes especially when autoantibodies, such as rheumatoid factor (RF) and anti-citrullinated peptide antibodies (ACPA), are present [2].

Autoantibody-positive RA has been historically associated with a more aggressive course, particularly in terms of radiographic progression, disability, and mortality. Together with this, data from large early arthritis cohorts have demonstrated that the 2010 American College of Rheumatology/European Alliance of Associations for Rheumatology (ACR/EULAR) classification criteria are less capable of identifying patients with autoantibody-negative RA [3]. The consequence of these observations, together with the greater diagnostic reliability of seropositive forms, is that the randomized clinical trials developed both in the past and recent years have mainly enrolled patients with seropositive disease (Table 1). Besides a lower inclusion in clinical trials, it was assumed that the response to therapy in patients with seronegative RA would be comparable to that of autoantibody-positive subjects. However, growing evidence suggests that the two serotypes of RA might significantly differ from various perspectives, not only pathobiological and clinical but also regarding outcomes [4]. A large study from the Leiden Early Arthritis Cohort evaluated the long-term outcomes of treatments among patients with RA and observed that the benefits from a treat-to-target regimen in improving disability and mortality and increasing the chance to achieve drug-free remission were present in autoantibody-positive patients only [2]. Further, despite being less aggressive in terms of radiographic damage, seronegative RA still shows high levels of disability and impairment of quality of life, as recently emerged in a study from three large early arthritis cohorts [5]. Post hoc analyses of clinical trials have also addressed significant differences between the two RA serotypes highlighting generally better responses in autoantibody-positive patients both for conventional and targeted therapies [6], at least partially underpinned by distinct pathobiological profiles within the synovial tissue [7]. In this regard, there are growing emerging data that characterize seropositive RA, seronegative RA, and other types of seronegative inflammatory arthritis differently at the histological level [8, 9].

**Table 1:** Percentage of seropositive patients enrolled in the main randomized clinical trials over the past 10 years

	b/tsDMARD	Author (year); journal	Study	% of seropositivity for RF and/or ACPA
*JAK-inhibitors*	Filgotinib	Combe B (2021); ARD [10]	FINCH 1	74–80
		Westhovens R (2021); ARD [11]	FINCH 3	75–78
		Genovese MC (2019); ARD [12]	FINCH 2	57–67^**+**^
		Westhovens R (2017); ARD [13]	DARWIN 1	67–84
		Kavanaugh A (2017); ARD [14]	DARWIN 2	72–83
	Upadacitinib	Rubbert-Roth A (2020); NEJM [15]	SELECT-CHOICE	62–65^**+**^
		van Vollenhoven R (2020); Arth & Rheum [16]	SELECT-EARLY	81–88
		Smolen JS (2019); Lancet [17]	SELECT-MONOTHERAPY	78–80
		Fleischmann R (2019); Arth&Rheum [18]	SELECT-COMPARE	87–88
		Burmester GR (2018); Lancet [19]	SELECT-NEXT	75–83
		Genovese MC (2018); Lancet [20]	SELECT-BEYOND	76–80
	Tofacitinib	Fleischmann R (2017); Lancet [21]	ORAL Strategy	78–75
	Baricitinib	Taylor PC (2017); NEJM [22]	RA-BEAM	87–92
		Fleischmann R (2017); Arth&Rheum [23]	RA-BEGIN	89–97
		Dougados M (2017); ARD [24]	RA-BUILD	72–77
		Genovese MC (2016); NEJM [25]	RA-BEACON	67–74
*TFNα-inhibitors*	Golimumab	Weinblatt ME (2014); ARD [26]	GO-FURTHER	100
	Certolizumab pegol	Ostergaard M (2023); ARD [27]	NORD-STAR	72–83
		Yamamoto K (2015); Mod Rheum [28]	HIKARI	85–89
		Smolen JS (2015; ARD [29]	CERTAIN	67–100
		Yamamoto K (2014); Mod Rheum [30]	J-RAPID	86–90
*B-cells depletion therapy*	Rituximab	Humby F (2021); Lancet [31]	R4RA	85–89
*Lymphocyte co-stimulation inhibitor*	Abatacept	Ostergaard M (2023); ARD [27]	NORD-STAR	72–83
*IL6-inhibitors*	Tocilizumab	Ostergaard M. (2023); ARD [27]	NORD-STAR	72–83
		Humby F (2021); Lancet [31]	R4RA	85–89
		Burmester G (2016); ARD [32]	FUNCTION	86–90
		Kivitz A (2014); Arth Care Res [33]	BREVACTA	81–84
	Olokizumab	Smolen JS (2022); NEJM [34]	CREDO 2	74–77
		Nasonov E (2021); ARD [35]	CREDO 1	80–89
	Sarilumab	Burmester GR (2017); ARD [36]	MONARCH	65–77
		Huizinga TWJ (2014); ARD [37]	SARIL-RA-MOBILITY	67–95

*b/tsDMARD* biologic/target synthetic disease-modifying anti-rheumatic drug, *RF* rheumatoid factor, *ACPA* anti-citrullinated peptide antibodies, *JAK* Janus kinase, *TNFα* tumor necrosis factor α

^**+**^Positive for both RF and ACPA

A similar trend is also emerging for the novel concept of the “pre-arthritis” stages, such as clinically suspect arthralgia and “arthralgia-at-risk” [38]. The concept of the “window of opportunity” has been indeed studied and extended in the context of inflammatory arthralgia without clinical arthritis. Several randomized clinical studies are currently ongoing, some of them showing promising results [39], in the so-called “preventive trials” aimed at evaluating the ability to halt the development of arthritis. This was conceptualized by the improved knowledge on RA pathogenesis, especially by the demonstration that autoantibodies—particularly ACPA—can be tracked in the sera of patients several years before the onset of clinical arthritis [40]. As a consequence, the ongoing randomized trials are again primarily tailored to autoantibody-positive patients which, however, are known not to represent the entire “at-risk” population [39]. The APIPPRA and ARIAA trials [41, 42] demonstrated that T-cell co-stimulation modulation with abatacept can delay, but not prevent, the development of RA and reduce the inflammatory burden over 2 years in autoantibody-positive subjects. A similar result was observed by the use of B-cell-depleting therapy in the seropositive pre-arthritis stages [43]. Conventional synthetic disease-modifying anti-rheumatic drugs (csDMARDs), hydroxychloroquine, and glucocorticoids have also been tested only in the “at-risk” population positive for autoantibodies [44, 45]. Furthermore, RF- and ACPA-positive subjects with arthralgia were the only population selected to determine the effects of a 36-month course of atorvastatin [46], or a plant-based diet combined with physical activity and mindfulness strategy [47] on the development of RA. One important exception is represented by the TREAT EARLIER trial, which included both seropositive and seronegative subjects with arthralgia clinically suspected of progressing to RA [48]. Although the trial failed to demonstrate a reduction in progression from the clinically suspect arthralgia stage to clinical arthritis, data from a post hoc analysis recently presented at the EULAR Congress 2024 showed that the greatest benefit of a very early intervention with methotrexate in order to prevent RA development might be particularly evident in ACPA-negative subjects [49]. Increasing evidence indeed indicates that the two serotypes of the disease may differ even during the preclinical phase [50]. ACPA-positive subjects have more often extra-synovial inflammation compared to ACPA-negative subjects in a magnetic resonance imaging study from the Leiden Clinically Suspect Arthralgia cohort, although developing RA to a similar extent [51]. Also, proteomic analyses have shown that multiple serum biomarkers identify imminent cases of RA, but only in ACPA-positive subjects [52]. Finally, an important consideration is that similar to what was observed in the most recent early arthritis cohorts which include more than 50% seronegative subjects [53–55], who still suffer from a non-neglectable diagnostic delay [56] and a considerable disease burden [53], this trend seems to be replicated in patients with clinically suspect arthralgia too [57, 58].

In conclusion, randomized clinical trials have primarily focused on autoantibody-positive RA patients, and a similar attitude is emerging in prevention trials. At the same time, critical unmet needs are arising from the population of seronegative patients. Broader inclusion into clinical studies and the development of tailored trials ultimately aimed at reducing the burden of the disease in autoantibody-negative patients are therefore needed.

## Data Availability

Not applicable.
